# Regulatory T Cells: Regulation of Identity and Function

**DOI:** 10.3389/fimmu.2021.750542

**Published:** 2021-10-05

**Authors:** Payal Grover, Peeyush N. Goel, Mark I. Greene

**Affiliations:** Department of Pathology and Laboratory Medicine, Perelman School of Medicine, University of Pennsylvania, Philadelphia, PA, United States

**Keywords:** Treg-regulatory T cells, FOXP3, T-effector cells, immunosuppression mechanisms, Tip60, autoimmune, antitumor immunity

## Abstract

T regulatory cells suppress a variety of immune responses to self-antigens and play a role in peripheral tolerance maintenance by limiting autoimmune disorders, and other pathological immune responses such as limiting immune reactivity to oncoprotein encoded antigens. Forkhead box P3 (FOXP3) expression is required for Treg stability and affects functional activity. Mutations in the master regulator FOXP3 and related components have been linked to autoimmune diseases in humans, such as IPEX, and a scurfy-like phenotype in mice. Several lines of evidence indicate that Treg use a variety of immunosuppressive mechanisms to limit an immune response by targeting effector cells, including secretion of immunoregulatory cytokines, granzyme/perforin-mediated cell cytolysis, metabolic perturbation, directing the maturation and function of antigen-presenting cells (APC) and secretion of extracellular vesicles for the development of immunological tolerance. In this review, several regulatory mechanisms have been highlighted and discussed.

## Introduction: A Historical Perspective

The immune system maintains a pool of Regulatory T cells (Tregs), a discrete population of CD4+ lymphocytes that regulates both innate and adaptive immune responses towards self-antigens, virulent agents, cancer, commensal microbiota, and various other allergens (1–5). Tregs play a prominent role in the maintenance of immune tolerance and normal functioning of the immune system or immune homeostasis by eliminating the autoreactive T cells, induction of self-tolerance, and curbing inflammatory processes (6–10).

The suppressive function of a class of T cells was first reported by Gershon and Kondo in the 1970s (11). Gershon’s laboratory discovered that the negative interference exerted by T cells during inflammation was distinct from helper T cells (T_H_ cells), hypothesizing that T cells not only enhanced but also weakened immune responses by downregulation of some biological functions (11). This suppressor T cell field was interrupted in the mid-1980s when analysis of mouse MHC gene failed to define the I-J DNA region, presumed to encode a molecule or domain of a molecule associated with their suppressive functions (12–14).

Other reasons that diminished interest in Regulatory cells was the inadequacy of specific markers for differentiating Tregs from other T cell populations, uncertainty in the molecular features of suppression, and difficulty in developing antigen-specific suppressor T-cell clones (13, 15). However, in the 1990s, the notion of T cell-mediated suppression resurfaced when a novel subgroup of CD4+ T cells were distinguished that co-expressed the interleukin-2 receptor (IL-2R) alpha-chain (CD25) and its ability to suppress autoimmunity in thymectomized mice (16).

Researchers have investigated the relationship between Tregs and tumors in the tumor microenvironment. Treg participation in anti-tumor immunity was first discovered in 1976 by Fujimoto and Greene (17). In 1999, others showed that in T cell-deficient animals transplanted with CD25+ cell-depleted splenocytes, anti-CD25 Ab depleting CD4+CD25+ Tregs inhibited tumor development (18). Reports have shown that Tregs infiltrate tumors and suppress the function of various immune cells including CD4+ T helper cells, CD8+ cytotoxic T cells, NK cells, and NK T cells (19–21). Thus, Tregs are able to suppress antitumor immunity, enabling tumors to progress faster, and their presence in the tumor microenvironment is directly correlated to a poor prognosis (22).

In humans roughly, 5-10% of the CD4+ T cell population in peripheral blood are comprised of naturally arising Tregs characterized by constitutive expression of CD25 and Forkhead Box 3 (FOXP3) (23–25). Mutations in FOXP3 were discovered to be associated with an autoimmune lymphoproliferative illness in humans termed X-linked autoimmunity-allergic dysregulation syndrome (XLAAD), which was later renamed Immunodysregulation, polyendocrinopathy, enteropathy, and X-linked (IPEX) syndrome (26). IPEX is one of the most well-known Mendelian disorders, characterized by a loss of immunological tolerance caused by a lack of functioning Treg cells (27, 28). As a result of these discoveries, effective Treg suppression is clearly necessary to avoid autoimmune and chronic inflammatory diseases.

## FOXP3: The Master Regulator of Tregs

Expression of FOXP3 serves as a dominant regulatory pathway in Treg development and function and is vital for Treg cell lineage identity (9, 29). FOXP3 is a member of the forkhead/winged-helix family of transcriptional factors. The gene is located on the X -chromosome and is highly conserved among different species. The FOXP3 protein is a 431 amino acid structure that is encoded by the *FOXP3* gene in humans. The protein has four domains: an amino-terminal proline-rich domain mediating transcriptional repression, a central zinc finger, and leucine zipper domains facilitating homo or hereto-dimerization and the C-terminal forkhead domain implicated in nuclear localization and DNA binding activity.

The N-terminal domain has a typical role in the development and function of Tregs (30, 31). The role of foxp3 in modulating immune tolerance was recognized by the discovery of “scurfy” mice exemplified by multi-organ lymphocytic infiltration, associated with mutations in the *foxp3* gene (32). In humans, the scurfy phenotype shared molecular and clinical features with IPEX, which was later linked to the human orthologous *FOXP3* gene (33).

Tregs appear more resistant to thymic deletion processes and are generated from the thymus during the early stages of fetal development (19). Treg cell activation is antigen-specific, inferring that Treg cells suppressive functions are antigen-dependent. Although self-reactivity of Treg cells has been proposed, extensive TCR repertoire analyses have revealed that self-reactivity is more likely to be the exception instead of the rule (34, 35).

A fraction of CD4+ expressing thymocytes/progenitor cells can differentiate into CD4+ CD25+ FOXP3+ T cells, commonly described as thymus derived Treg (or tTreg), or natural Treg (nTreg). Treg cells can also be generated extrathymically following antigenic stimulation of conventional CD4+ T cells/naive T cells at peripheral sites (lymphoid or non-lymphoid tissues) and therefore are designated as the peripheral (pTreg) Treg cells (*in vivo*). Upon stimulation with anti-CD3 in the presence of cytokines such as TGF- β and IL-10, induced Tregs (iTreg) cells (*in vitro*) can be established in cell cultures (36–38). While some investigations have also shown that Helios and neuropilin-1 (Nrp1), a semaphorin III receptor, can be used to identify tTreg and pTreg cells, there are several instances that clearly do not distinguish between thymus and extrathymically derived Tregs (39–41). Even though no one marker distinguishes these three distinct subgroups of Tregs, FOXP3 expression is indispensable for their suppressive function.

Studies have conjointly indicated that tTreg cells can develop into specialized effector Treg subsets, such as tissue resident Treg cells that play a significant role in non-lymphoid organ function (42, 43). Tregs in visceral adipose tissue, muscle Tregs, and skin-resident memory Tregs have been described emphasizing some intriguing biological implications about their functional interactions with local tissues (43–47).

FOXP3 interacts with various transcriptional and chromatin-modifying factors and acts as a “master” regulator of Treg cell development process (9, 48, 49). FOXP3 can modulate the transcriptome of Treg cells by various mechanisms depending on binding partners. FOXP3 forms large protein complexes ranging from 300 KDa to over 12000 KDa encompassing many proteins that have either a direct or indirect interaction within this interactome *per se* (50, 51).

Binding of AML/Runx1 (Acute myeloid leukemia 1/Runt-related transcription factor 1) to FOXP3 leads to upregulation of Treg-related molecules by repressing IL-2 and IFN-γ levels (52). Runx1 also forms a complex with core-binding factor subunit beta (CBFβ) and precisely adheres to the FOXP3 promoter region’s conserved non-coding sequencing region 2 (CNS2), which is significantly de-methylated in Tregs and is required for FOXP3 expression and Treg cell lineage stability (53). Interaction of FOXP3 with the nuclear factor of activated T cells (NFAT) complements the expression of CD25, cytotoxic T-lymphocyte-associated protein 4 (CTLA4), and glucocorticoid-induced TNF receptor (GITR) while curbing the expression of inflammatory genes IL-2 and IFN-γ, serving as a transcriptional activator of Treg cells (54–56). NFAT is required by both iTregs and pTregs, but not by tTregs. NFAT binds to FOXP3 and suppresses the expression of NFAT-targeted genes. NF-κβ molecules Rels (both RelA and c-Rel) have a role in pTreg formation as well as FOXP3-regulated gene expression and repression (57, 58). RelA deletion, which is Foxp3-specific, causes more severe autoimmune symptoms than c-Rel deletion, indicating that RelA is more essential than c-Rel in Treg function (58).

Additionally, Eos (IKZF4), GATA3, Interferon regulatory factor 4 (IRF4), signal transducer and activator of transcription (STAT3), Retinoic acid receptor-related orphan receptor (RORγT), RORα, YY1, hypoxia-inducible factor 1-alpha (HIF1α), FOXO1, FOXO3, and Satb1 are among the transcription factors that have been reported to interact with FOXP3 to promote Treg identity (50, 59–67). As a result of these numerous descriptions, it has been shown that the presence of these components upstream of *FOXP3* orchestrates Treg lineage and makes FOXP3 indispensable for Treg suppressor activity.

The function of FOXP3 can also be regulated by several posttranslational modifications (PTMs) including acetylation, phosphorylation, ubiquitination, and methylation (29). These PTMs govern its DNA binding capability, stability, and protein-protein interactions (transcriptional co-activators, transcriptional repressors, and chromatin remodelers) that modulate Treg suppressive functions. The process of acetylation involves acetylation and deacetylation of specific lysine residues catalyzed by the activities of both Histone acetyltransferases (HATs)/lysine acetyltransferases (KATs) and Histone deacetylases (HDACs). The principal acetylase include Tip60 (KAT5), a member of the MYST family, and p300 (KAT3b) belonging to the p300/CBP family (68, 69). Both Tip60 and p300 act cooperatively to acetylate FOXP3 (70).

p300 interacts with Tip60 to stimulate auto-acetylation and this interaction affects the stability of Tip60. Following this interaction, p300 acetylates the K327 of Tip60, which changes its substrate interaction and acts as a “molecular switch”, permitting Tip60 to change binding partners. Because of this, Tip60 associates with FOXP3, acetylates FOXP3 leading to release of p300. Throughout these events, Tip60 itself acetylates p300, which is indispensable for its HAT activity. Synergistic actions of both Tip60 and p300 contribute to maximal stimulation of FOXP3 repressive transcriptional activity and enhanced stability by preventing its polyubiquitylation-mediated degradation (70, 71).

In an earlier study from our laboratory, we first showed that Tip60, HDA7, and HDAC9 were associated with FOXP3 in a dynamic ensemble forming a chromatin remodeling complex where Tip60 constituted as a vital subunit in the repression complex. We found that FOXP3 exerted higher repression in the presence of over-expressed Tip60 levels when compared to its HAT deficient mutant forms. Further, the knockdown of Tip60 eased transcriptional repression (69). In subsequent studies from our laboratory, we showed that the conditional knockout of Tip60, but not p300 *in vivo*, leads to scurfy like an autoimmune disease by significantly lowering the Treg cell population in spleen and lymph nodes (70). Further, naïve CD4+ T cells transduced with foxp3 and Tip60 mutants (Q377/G380E and K327Q) exhibited lowered Treg suppressive function compared to foxp3 and wild type (WT) Tip60.

**Figure 1:**
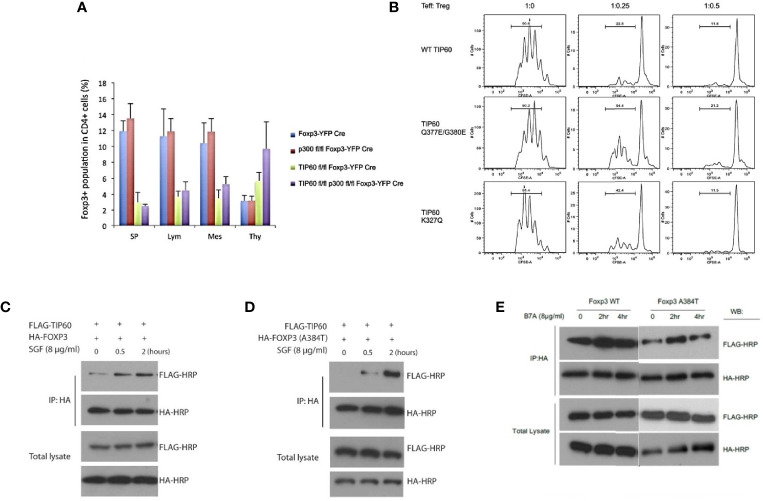
Importance of Tip60 in maintenance of peripheral Treg cell population [Figure adapted from (70, 71)]. **(A)** Average percentage of Treg cell populations from Foxp3^YFP-Cre^, p300^fl/fl^ Foxp3^YFP-Cre^, Tip60^fl/fl^ Foxp3^YFP-Cre^ and p300^fl/fl^ Tip60^fl/fl^ Foxp3^YFP-Cre^ mice in spleen, lymph nodes, mesenteric lymph and thymus **(B)** CD4+ T cells transduced with Foxp3 and WT TiP60 or TiP60 mutants (Q377/G380E and K327Q) **(C–E)** Effect of allosteric modifiers (SGF003 and B7A) targeting Tip60 help stabilize Tip60-FOXP3 interaction and restoring Treg suppressive capacity. Here, in the presence or absence of SGF003 (8 μg/mL), 293T cells were transfected with HA-FOXP3 (WT) and HA-FOXP3 (A384T) and FLAG-TIP60, or HA-FOXP3 (WT) and FLAG-TIP60, in the presence or absence of B7A (8 μg/mL). Cells were washed with PBS 24 hours post transfection, and cell lysates were extracted for immunoprecipitation and western blot analysis. The effects of SGF and B7A treatment on the interaction of FOXP3 with the flagged proteins are represented.

In disease processes such as IPEX, one of the most common mutations (A384T) occurring in the C-terminal forkhead domain disrupts the Tip60-FOXP3 interaction affecting Treg functions. Our laboratory has identified small allosteric modifiers (SGF003 and B7A) that are able to target Tip60. We employed a cavity-induced allosteric modification (CIAM) approach that can help stabilize Tip60-FOXP3 interaction and can restore Treg suppressive capacity (71, 72). These observations identify some of the important roles of Tip60 in Treg biology [**Figure 1**, adapted from (70, 71)].

Methylation of FOXP3 occurs on arginine residues by the activity of Protein methyl transferases (PRMTs) exerts a pivotal role in Treg function. PRMT5 is the core type II protein arginine methyltransferases found abundant in all eukaryotic species (73, 74). In recent studies, it was demonstrated that the cholesterol biosynthesis metabolic gene expression program that induces RORγt agonistic activity and promotes Th17 differentiation and experimental autoimmune encephalomyelitis is dependent on “PRMT5 expression” in newly activated T cells (75). Additionally, recruitment of STAT3 by PRMT surmounted the inhibition of Th17 differentiation mediated by STAT5 activation induced by IL-2. As a result, PRMT influenced Th17 differentiation by controlling the reciprocal recruitment of STAT3 and STAT5. Identifying PRMT members that act as a potential target for reducing RORγt-dependent production of pathogenic Th17 cells may be relevant to therapeutics that may relieve Th17-mediated autoimmunity (76).

Conditional deletion of PRMT5 in Treg cells alters their quantity and function, resulting in a scurfy phenotype, according to recent data from our laboratory. We found that in Foxp3+ cells, silencing PRMT5 resulted in reduced suppressive activity, which affected the relevance of PRMT5 in Treg function maintenance. Inhibiting PRMT5 affected anti-tumor immunity by limiting the infiltration of Treg cells into tumor sites. In a syngeneic mouse model of breast cancer (erbB2/neu), we reported that DS-437, a PRMT5 inhibitor, dramatically improved anti-HER2/neu therapy in rodents (77). Taken together, these findings point to the critical functions of PRMT5 and PRMT1 in regulating FOXP3 function by regulating Treg cell activity and function.

## Suppressive Mechanisms of Treg Cells

**Figure 2:**
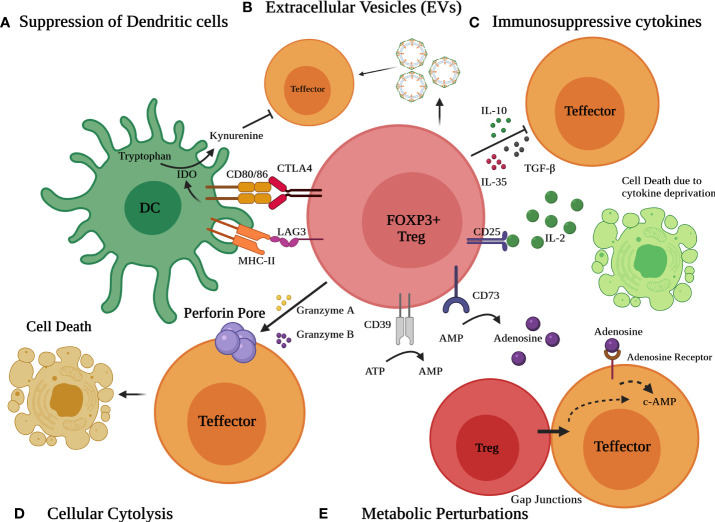
Different modes of Immunosuppression by Treg cells. Various mechanisms include **(A)** suppression of dendritic cells (DCs) to modulate their maturation and function *via* CTLA4 and LAG3 **(B)** release of extracellular vesicles (EVs) **(C)** secretion of suppressive immunoregulatory cytokines such as TGF-β, IL-10, and IL-35 **(D)** Granzyme/perforin mediated cellular cytolysis and **(E)** metabolic perturbations involving CD25 dependent cytokine deprivation, generation of adenosine by ectoenzymes CD39 and CD73 and c-AMP mediated inhibition.

Tregs have the potential to alter immune function in cells of both the innate and adaptive immune systems. Treg cells suppress a myriad of immune cells, notably B cells, CD4+ T cells, CD8+ cytotoxic T cells, NK cells, NKT cells, macrophages, dendritic cells, neutrophils, and T cells (1, 78). Tregs use a variety of immunosuppressive methods to dampen immune responses (**Figure 2**). These include (a) immunosuppressive cytokines such as TGF-β, IL-10, and IL-35 (b) Metabolic perturbations involving CD25 (IL-2 receptor alpha) dependent cytokine deprivation facilitated apoptosis, immunosuppressive adenosine by ectoenzymes CD39 and CD73 and c-AMP mediated inhibition, (c) granzyme and perforin mediated cytolysis (d) Interaction with antigen-presenting cells (APC) such as dendritic cells (DCs) to modulate their maturation and function (CTLA4 and LAG3) and (e) extracellular vesicles (EVs) generated from Tregs (8, 9, 24, 78–83).

Distinct processes may function in different situations, with one taking precedence in one process and the other in the another. Alternatively, multiple suppressive mechanisms act in concert and may do so synergistically, such that limiting of any one of these pathways not enough to compromise suppression activity significantly (84).

### Inhibitory or Immunosuppressive Cytokines

Suppressive effects mediated by Treg cells can be achieved *via* the release of soluble mediators or Treg-associated cytokines such as TGF-β, IL-10, and IL-35 (member of IL-12 family) (79, 85). The main mechanisms of action of these cytokines are (a) Restrict stimulation and/or survival of effector T (Teff) cells in the state of autoimmunity by inhibiting the autoreactive Teff cell activation and (b) Production of iTreg cells supporting peripheral homeostasis and survival of these cells (24).

Treg cells produce a significant amount of both soluble and membrane-bound TGF- β, and blockage of TGF- β using anti-TGF- β impairs T cell proliferation *in vitro* (both murine and human T cells) (86). In another study, mice lacking TGF-β were shown to develop chronic autoimmunity and had low levels of CD4+ CD25+ Treg cells following 4 to 5 weeks (87, 88). In the presence of TGF-neutralizing antibodies, both murine and human Treg responses were found to be impaired, emphasizing the relevance of this immunosuppressive cytokine (89). In a recent study, RING-type E3 ligase Arkadia, that governs TGF- β signaling during development, was shown to be required for iTreg but not Th17 and its ablation in T cells resulted in a higher propensity to inflammatory bowel disease (90). In yet another study, it was found that monoallelic deletion of *Tgfb1* in Treg cells resulted in allergic response by impairing RORγT mediated Treg development. *Tgfb1* deletion caused catastrophic autoimmunity with a scurfy-like phenotype involving autoantibody synthesis and impaired follicular T helper and B cell responses in Treg cells (91). Together these observations highlight the role of TGF-β in both allergic and autoimmune responses.

IL-10 production by Treg cells appears to play an important role in regulating intestinal inflammation by regulating tolerance towards commensal microbiota. Mice lacking IL-10 in Tregs are highly susceptible to colitis using Inflammatory bowel disease (IBD) models and exhibit immune reactivity in the airways (92, 93). On a similar note, Treg cells lacking IL-10R in mice resulted in dysregulation of Th17 cell responses and development of colitis by inhibition of STAT3 signaling (94). Likewise, mutations in genes encoding IL-10 and/or IL-10R are associated with Crohn’s disease and ulcerative colitis in humans (95). IL-10 also plays an important role in tumor development. The tumor microenvironment (TME) promotes the generation of Treg cells that mediate IL-10 dependent immune suppression in a cell-contact independent manner. However, the suppression activity was abolished in the presence of neutralizing IL-10 antibodies (96). Similarly, IL-10 is also involved in antitumoral immunity mediated by UV-induced carcinogenesis in mice (97).

IL-35 is a heterodimeric cytokine that belongs to the IL-12 family and has been implicated in some studies of Treg mediated suppression to act as a novel inhibitory cytokine (85). IL-35 is composed of the IL-12a chain (p35) and Epstein Barr virus gene 3 (EBI3) product. Treg cells impaired in either chain of the dimeric IL-35 possess reduced suppressive activity and are incapable of curbing IBD and homeostatic T cell expansion symbolizing the role of IL-35 in Treg suppressive functions *in vivo* (85). Moreover, IL-35 has been suggested to play a role in human immunosuppression by suppressing the proliferation of T cells and promoting the conversion of naive T cells to induce regulatory T cells (iTr35) without the requirement of IL-10, TGF-β, or FOXP3 (98) affirming the role of IL-35 in human Treg function.

Another study discovered the effector population of Tregs that produced IL-35 differed from that of Tregs that produced IL-10. Blimp1 was found necessary for IL-10 production but not for IL-35, whereas Foxp3 was required for IL-35 but not for IL-10. Therefore, the TCR signal influences the discrete synthesis of IL-35 and IL-10 during the generation of effector Tregs, implicating a mechanism of differential cytokine expression that permits Treg functional characteristics to be tailored to a range of immunological responses (99).

These cytokines modulate Treg functions and are associated in the polarization of immune responses in a variety of diseases.

### Metabolic Disruption

Metabolic effects are employed by Treg to inhibit the immune response involves. These effects include competition for IL-2, repression by c-AMP and CD39, and/or CD73 generated adenosine receptor 2A mediated immunosuppression.

IL-2 promotes the survival and proliferation of T cells (100). IL-2R is formed of α (CD25), β (CD122), and γ (CD132) subunits where IL-2Rα increases the affinity towards IL-2. Treg cells express higher levels of CD25 and thus may possess a higher affinity towards IL-2, competing with proliferating cells (101, 102). Thus, by limiting the IL-2 levels, Treg cells thwart the stimulation of Teff cells in the periphery, triggering metabolic disturbance culminating in cellular apoptosis (103). In one of the studies, it was shown IL-2 signaling is requisite for Treg suppressor function through STAT5b activation (104).

The concordant expression of ectoenzyme ATP apyrase (CD39) and ecto-5’- AMP-nucleotidase (CD73) suppresses Teff cell functions by activation of adenosine receptor 2A (A2A) generates the purine nucleoside Adenosine (105). FOXP3 regulates the expression of CD39 in Treg cells that break down ATP to AMP and later CD73 rapidly degrades AMP to adenosine. The adenosine receptor (A2A) expressed on T cells is tightly coupled to G-protein-coupled receptor (GPCR) that mediates an increase in c-AMP following adenosine signals to mediate inhibitory signaling (106).

Treg cells can also transfer c-AMP to the Teff cells, a process that involves membrane gap junctions where these secondary messengers can activate c-AMP dependent protein kinase A (PKA) (107, 108). Activation of PKA leads to phosphorylation of C-terminal Src Kinase (CSK) that negatively regulates another Src Family Tyrosine Kinase (LCK), involved in the proximal activation of T cell receptors by downregulation of T cell receptor (TCR) signaling (107, 109). PKA also phosphorylate cAMP Response Element-Binding protein (CREB) that regulates the expression of Foxp3 required for the development and function of Tregs (110, 111).

Studies from our laboratory have shown that the transcription of Foxp3 and Treg development is based on the formation of “c-Rel enhanceosome” that contains CREB, p65, NFAT, and Smad3 providing an additional instance of CREB inhibiting immune system (112). These findings suggest that elevation in intracellular c-AMP following A2A activation optimizes Treg activity leading to suppression of Teff cells.

### Cellular Cytolysis

Treg cells are known to suppress target cells by a cytolysis-like process involving granzymes in both humans and mice (113, 114). Use of granzyme/perforin-mediated cytolysis by NK and CD8+ cytotoxic T cells to eliminate virus-infected and tumor cells has long been recognized (115).

Exocytosis involves the extracellular space at the Treg-Teff cell interface, mediated by Treg cells as granules containing perforins and granzymes. These perforin glycoprotein molecules attach to the target cell’s plasma membrane, polymerize, and produce pores that permit granzyme movement (116). Granzymes also use receptor-mediated endocytosis to kill target cells by interacting to the mannose-6-phosphate receptor (117).

Mice with Granzyme B knockout Treg were used for the first study to verify that Treg cells possess cytolytic potential. Granzyme deficient Treg cells have a diminished suppressive capability (118). Treg cells also inhibit B-cell proliferation by eliciting B-cell death through a granzyme-dependent but partially perforin-dependent mechanism (119). Finally, in later set of experiments Treg cells were found to be capable of suppressing both NK and CD8+ cytotoxic T cell-mediated antitumor responses in a granzyme-B and perforin-dependent manner (120). Treg express Granzyme A and may eliminate autologous target cells in a perforin-dependent fashion (113).

These studies indicate that Treg may act directly on B cells, implying that the direct control of B-cell activity is at least partially responsible for Treg’s potential to suppress autoimmunity. Furthermore, the perforin-granzyme pathway is crucial not only for NK and CD8+ T cell function, but it may also be used by Treg cells to restrict these cells’ activity. Thus, the perforin/granzyme pathway appears to be one of the ways that Treg cells utilize to regulate immunological responses. Hence, cytolysis may allow Treg cells to confine the number of effector cells and contain an immune response.

### Suppression of Antigen Presentation

APC interactions with Treg cell at the immune synapse has relevance toimmune suppression. This interaction may alter the maturation and function of DCs *via* contact-dependent mechanisms by influencing the DCs costimulatory ability required for the activation of Teff cells. One of the well-recognized interaction sites involves CTLA4, the co-stimulatory molecule constitutively expressed by Treg with CD80/CD86 expressed on DCs (121, 122). This process leads to the production of Indoleamine 2,3 dioxygenase (IDO) that catalyzes the catabolism of essential amino acid tryptophan to kynurenine. The scarcity of tryptophan suppresses the protein synthesis resulting in cell cycle arrest and thus inactivity or anergy of Teff cells (101, 123, 124). Wing K et al., found that mice bearing Treg-specific deletion of CTLA4 develop systemic and fatal autoimmune lymphoproliferative disease (125). These results were further corroborated when a similar phenotype was observed in humans with heterozygous mutations in CTLA4 (126). These CTLA4 is needed for immune suppression and plays a key role in both biological and pathological immune responses.

Additionally, a CD4-related protein expressed on the surface of Tregs, Lymphocyte-activation gene 3 (LAG3 or CD223), binds to MHC-II molecules on DCs with high affinity and blocks their maturation. It should be noted that despite its greater affinity for MHC-II:CD4, LAG3 interferes with TCR but has no effect on MHC-II:CD4 interaction (127). This binding activates immunoreceptor tyrosine-based activation motif (ITAM) mediated inhibitory signaling that involves activation of extracellular-signal-regulated kinase (ERK) and recruitment of protein tyrosine phosphatase 1 (SHP1) (128, 129). Together these processes further suppress DC maturation and the antigen-presenting/immunostimulatory ability of DCs. Hence, Treg cells target the maturation and costimulatory functions of DCs.

### Immune Tolerance Is Affected by Generation of Extracellular Vesicles (EVs)

EVs are membranous structures produced from cells that have a role in physiologic and pathologic processes. EVs generated from Treg cells have been shown in several studies to be a precise intercellular exchange mechanism governing immunological responses and therefore establishing a tolerogenic milieu in a cell-free manner. The hypothesized pathways include miRNA-induced gene silencing, surface protein activity, and enzyme transfer. Treg cells may undergo transformation into effector T cells following exposure to inflammatory conditions lends credence to these findings. EVs, on the other hand, are unlikely to be changed under inflammatory circumstances, in contrast to their cells of origin (82).

Tregs have also been found to communicate with one another: intercellularly by releasing small extracellular vesicles (EVs). Following TCR activation, CD4+CD25+ Tregs have been shown to release EVs in rodents and human settings. These vesicles exhibit immunological modulatory abilities *in vitro*, comparable to the cell from which they were originated (81–83). Murine Treg EVs have been shown to reduce CD4+ Teff cell proliferation, as well as IL-2 and IFNγ release, and enhance IL-10 production by murine DCs in recent studies. This process has been ascribed to the cell surface immune modulatory molecule CD73, an ecto enzyme implicated in adenosine synthesis, as well as miRNAs found in these vesicles, such as miR-142 and miR-150 (83, 130). EVs generated from Treg cells have been found able to inhibit T cell-mediated responses by transferring micro-RNAs, namely miR-155, Let-7b, and Let-7d RNAs. While isolated Treg cells derived EVs could suppress conventional T cells, they were not as effective as Treg cells, indicating that additional processes are necessary for optimum suppression (131). These results raise a slew of new questions and possibilities about the involvement of Treg cells derived EVs in a variety of immunological settings.

## Conclusions and Future Perspectives

In the immune system, Treg cells perform both positive and negative roles. Immune suppression mechanisms to limit autoimmunity, transplantation and maintain immune homeostasis are positive effects, whereas circumvention of antitumor immunity is a negative effect. Tregs immunosuppressive activity in tumors environments represents a significant barrier to efficient anti-tumor immunity. As a result, research into the roles and activities of Tregs is needed to fully understand their potential as immunotherapeutic targets and to develop new tumor immunotherapy methods.

Treg biology is complex. Understanding the molecules and detailed structural and functional domains of regulatory proteins as well as signaling pathways in target cells affected by Treg may be needed for potential therapeutic interventions of effective immune response.

## Author Contributions

All authors contributed to data analysis, drafting or revising the article, gave final approval of the version to be published, agreed to the submitted journal, and agree to be accountable for all aspects of the work.

## Funding

This work was supported by grants from the National Institutes of Health (R01CA219034 and R21AI35359) and Breast Cancer Research Foundation (BCRF-17-061) to MG.

## Conflict of Interest

The authors declare that the research was conducted in the absence of any commercial or financial relationships that could be construed as a potential conflict of interest.

## Publisher’s Note

All claims expressed in this article are solely those of the authors and do not necessarily represent those of their affiliated organizations, or those of the publisher, the editors and the reviewers. Any product that may be evaluated in this article, or claim that may be made by its manufacturer, is not guaranteed or endorsed by the publisher.
